# Validation and Psychometric Properties of the Persian Version of the 21-Item Game Addiction Scale With a Sample of Adolescents and Young Adults

**DOI:** 10.3389/fpsyt.2021.649276

**Published:** 2021-05-24

**Authors:** Nasrin Abdoli, Vahid Farnia, Mostafa Alikhani, Dena Sadeghi-Bahmani, Kenneth M. Dürsteler, Maryam Esmaeili, Annette Brühl, Serge Brand

**Affiliations:** ^1^Substance Abuse Prevention Research Center, Health Institute, Kermanshah University of Medical Sciences, Kermanshah, Iran; ^2^Sleep Disorders Research Center, Kermanshah University of Medical Sciences, Kermanshah, Iran; ^3^Center for Affective, Stress and Sleep Disorders Zentrum fär Affektive-, Stress- und Schlafstörungen (ZASS), University of Basel, Psychiatric Clinics Universitäre Psychiatrische Kliniken (UPK), Basel, Switzerland; ^4^Departments of Physical Therapy, University of Alabama at Birmingham, Birmingham, AL, United States; ^5^Division of Substance Use Disorders Basel, University of Basel, Psychiatric Clinics, Basel, Switzerland; ^6^Department of Psychology, Faculty of Education, University of Isfahan, Isfahan, Iran; ^7^Division of Sport Science and Psychosocial Health, Department of Sport, Exercise, and Health, University of Basel, Basel, Switzerland; ^8^School of Medicine, Tehran University of Medical Sciences (TUMS), Tehran, Iran

**Keywords:** gaming, validation, game addiction, adolesents, adults, Persian, Farsi

## Abstract

**Background:** Excessive gaming has become a psychological health issue for both gamers and their social environments. This observation holds true for western but also non-western countries such as Iran. The aim of the present study was to translate and validate a Persian version of the Game Addiction Scale 21 (GAS 21) using a sample of adolescents and adults.

**Methods:** A total of 412 participants (mean age: 22.16 years; 55.3% females) took part in the study. They completed questionnaires covering sociodemographic information and gaming-related information, as well as the Persian version of the GAS 21, the GAS 7, the Internet Addiction Test, and the Generalized Self-Efficacy Scale.

**Results:** Of the initial 21 items of the Persian version of the GAS 21, five proved redundant and were eliminated. Factors analyses yielded four factors explaining 66.35% of the variance: 1. Withdrawal; 2. Feelings of guilt and addiction; 3. Mood modification; 4. Issues of time budget. Cronbach's alphas were satisfactory (alphas > 0.87). To validate the results, scores on the translated version were compared with the well-established Young Internet Addiction test. Factors correlated positively (rs between 0.21 and 0.31) with overall score on this latter test but, against expectations, positively with the generalized self-efficacy scale.

**Conclusions:** A Persian version of the Game Addiction Scale-21 proved to be a useful tool for assessing the risk of game addiction behavior. Further, the solution with 16 items loading on four factors appears respond to the ecological need of parsimony.

## Introduction

There is striking evidence that prolonged screen time is associated with negative psychological health outcomes for adolescents (1–4). These unfavorable associations are true not only for excessive and problematic smartphone use (2–4), but also for screen time in general. Internet addiction, understood as a proxy for excessive and pathological screen time, has for example been linked to suicidal behavior (5), generalized adverse psychological health outcomes (6), eating disorders (7), social anxiety disorders (8), and increased substance use (9).

**While Gambling disorder is listed as a recognized behavioral disorder in the DSM-5 (F63.0)** **(****10****); this is not the case for Internet Gaming Disorder, which has been just** ***tentatively* identified as disorder in the DSM-5. Or the other way around: it is still a matter of debate if (internet) gaming disorder should be classified as a psychiatric disorder as its own entity. As regards the ICD-10, behavioral disorders such as compulsive buying, working addiction, porno addiction, sports addiction, or internet addiction were classified as F63.8; impulse control disorder, with the specific specifier** **(****11****). As regards the ICD-11, the new category is labeled Disorders due to Addictive Behaviors (6C5), with the following subcategories: Gambling Disorder (6C50), Gaming Disorder (6C51), Other specified (6C5Y), and Unspecified (6C5Z). For Gaming and Gambling Disorder, the following specifiers are listed: Predominantly offline (6C50.0/6C51.0), Predominantly online (6C50.1/6C51.1), and Unspecified (6C50.Z/6C51.Z;** **https://www.who.int/classifications/classification-of-diseases****; retrieved March 31, 2021). Next, for** the following reasons Rumpf et al. (12) underscored the need to introduce Gaming Disorder as a psychiatric disorder as its own entity: 1. Excessive gaming has become an individual, public and clinical health concern. 2. In the meanwhile, there is sufficient data-based evidence that excessive gaming leads to dramatic health concerns. 3. The gaming industry should get aware to carry the responsibility for consequences of their products.

Excessive internet gaming (online and/or offline) is understood as continuous and repeated use of the internet to play games (either alone or with other people) within the last 12 months. Typically, and to name a few, individuals with excessive internet gaming report impairment and distress in several aspects of their life such as preoccupation with gaming, withdrawal symptoms when gaming is taken away or not possible (sadness, anxiety, irritability), continuing to game despite problems, deceiving family members or others about the amount of time spent on gaming, along with the risk of jeopardizing or losing a job or relationship due to gaming (10). It has been estimated that among adolescents the prevalence rates range from 4.5% (females) to 8.4% (males), with dramatically higher prevalence rates in Asian countries such as China and South Korea (10). Wartberg et al. (13) reported that 3.2% of a sample of 1,723 German adolescents fulfilled the criteria for pathological internet use, while Riedl et al. (14) reported the following prevalence rates for a sample of 298 adolescents in Austria: problematic internet use: 7.7%; pathological internet use: 3.3%; pathological computer game usage: 5.4%.

Overall, the scientific community agrees with the observation that behavioral addiction disorders are increasing worldwide and that such behavioral addiction disorders have the potential to become an economic, individual, and societal health issue.

Further, there is evidence that Internet addiction and Gaming addiction are two behavioral health issues with some conceptional overlaps, but also with clear distinctions. Ryding and Kaye (15) summarized in their overview, that Internet Addiction could be generally considered as an impulse disorder; typically, individuals with internet addiction experience intense preoccupation with using the internet, and, among others, a decreased social interaction in the real world. But Ryding and Kaye (15) also justifiably underlined that the internet is “simply” a means to get access to and to use a broad range of further activities such as professional information gathering (emailing; using scientific search engines such as Pubmed® or Psychinfo®), entertainment including gaming and gambling, online shopping, socializing (social network sites, group chats, dating platforms), just to mention a few. In the same vein, Starcevic and Aboujaoude (16) argued that Internet addiction is conceptually too heterogeneous and should be replaced by the specific behaviors (e.g., gaming, gambling, or sexual activity), irrespective from whether such specific behaviors are performed online or offline. Thus, given this background, we used the Young Internet Addiction Test (YIAT; see below) to validate the present Game Addiction Scale.

As regards the situation in Iran, there is increasing evidence that excessive screen time and excessive internet gaming have become major public health concerns. (17) reported that, of a sample of 4,261 university students, 27.3% indicated they suffered from problematic internet use. Problematic internet use was associated with other health-related issues such as substance use, suicidal ideation, and poor general health (17). Lin et al. (18) described four different studies of gaming addiction among adolescents; prevalence rates ranged from 5.3 to 17%. Not surprisingly, more problematic internet gaming was associated with poorer physical and mental health reflected in increased symptoms of anxiety and sleepiness and impaired social functioning.

A thorough literature search in Pubmed® and Psychinfo® showed that in Iran, problematic gaming has not been assessed across generations; either adolescent or adult samples have been assessed. While from a methodological point of view it might make sense to assess adolescents and young adults separately (19), from a neuro-developmental perspective such a differentiation is open to dispute. Giedd et al. (20–22) have shown in their longitudinal studies that brain maturation continues until about 25 years of age. Furthermore, as evidenced above, game addiction is not restricted to adolescence. In the present study, we therefore assessed gaming addiction from young adolescence to young adulthood.

Self-efficacy is a psychological and psychotherapeutic concept related to health behavior. It is understood as the belief that one can turn an idea or an intention into a successful achievement, and that such successful achievement can be fully explained by one's own engagement and performance (23). Unsurprisingly therefore, high self-efficacy has been associated with lower scores for gaming addiction (14, 24). Relatedly, Jalilian et al. (25) investigated the social and psychological determinants to predict the intention to use marijuana (or to refrain from marijuana use) and showed that higher self-efficacy and higher problem-solving skills predicted a lower intention to use marijuana. In the present study, we assessed the relation between self-efficacy and game addiction scores.

In brief, the aim of the present study was to investigate the psychometric properties of a Persian version of the Game Addiction Scale (GAS 21) and to relate scores on this measure to validated questionnaires on problematic internet use and generalized self-efficacy.

We believe that the present results should determine whether the Game Addiction Scale is appropriate for assessing problematic internet gaming behavior among Persian-speaking adolescents and adults. Specifically, it appears that no such Farsi/Persian measurement has been introduced and psychometrically assessed for a broader age range of internet users. This appears to hold particularly true for samples spanning a larger developmental range from adolescence to adulthood.

## Methods

### Study Design

To recruit participants, the study was advertised on the homepages of the Kermanshah University of Medical Sciences, on homepages of secondary and high schools of the city of Kermanshah (Iran), and on internet cafés and gaming halls. Participants between 12 and 40 years from Kermanshah City (Kermanshah, Iran) and self-reporting issues with gaming were invited to participate in this cross-sectional study on game addiction. The age range spans the developmental period of early adolescence (26) to the end of young adulthood (27, 28). Those eligible for participation were fully informed about the aim of the study and the anonymous data handling, and signed a written informed consent. Next, participants completed a Persian version of the Game Addiction Scale 21 (see below), a Persian version of the Game Addiction Scale Short Form (7 items; see below), the Persian General Self-efficacy Scale (see below), and the Internet Gaming Disorder Test (see below). Completion of the questionnaires took about 15 min. The ethical committee of the Kermanshah University of Medical Sciences (KUMS; Kermanshah, Iran) approved the study, which was performed in accordance with the ethical principles laid down in the seventh and current edition (29) of the Declaration of Helsinki.

### Participants

The initial sample consisted of 430 participants. Inclusion criteria were: 1. age between 12 and 40 years; 2. self-reported gaming time of 1 h a day and self-reported issues such as conflicts with their social environment, having difficulties stopping gaming, gaming longer than planned, and neglecting other tasks such as homework, household chores, or neglecting family members. 3. compliance with the study conditions; 4. willing and able to complete questionnaires in Persian; 5. signed written informed consent. On the basis of an initial inspection of the data, participants were excluded when they had completed the questionnaires within 5 min or less, or when only right or left boxes had been ticked, each of these suggesting that questionnaires were answered with low accuracy and low reliability. Eighteen participants were excluded on this basis. The final sample consisted of 412 participants (mean age: 22.16 years; 55.3% females; see also Table 1).

**Table 1:** Overview of sociodemographic descriptive and statistical indices, separately for male and female participants.

	**Gender**		**Statistics**
	**Females**	**Males**	
	*N* (%)	*N* (%)	
	221 (53.6)	191 (46.4)	
	M (SD)	M (SD)	
Age (years)	22.51 (10.43)	20.68 (9.21)	t(410) = 1.87, *p =* 0.62, d = 0.19 (T)
Gaming time/day (h)	4.02 (2.78)	4.63 (2.91)	t(410) = 2.18, *p =* 0.03, d = 0.21 (S)
BMI	22.88 (4.49)	24.19 (6.03)	t(410) = 2.43, *p =* 0.015, d = 0.23 (S)
	n (%)	n (%)	
Highest educational level (illiterate/compulsory school/high school diploma/higher education)	2/73/144/2	4/66/11/10	X^2^(N = 412, df = 3) = 8.48, *p =* 0.40 m
Current job position (unemployed/employed/student/worker/free-lancer)	19/19/106/44/33	23/15/94/26/33	X^2^(N = 412, df = 4) = 4.04, *p =* 0.41
Father had addiction behavior (yes/no)	18/203	12/179	X^2^(N = 412, df = 6) = 0.53, *p =* 0.47
Prevalent device of gaming (mobile/tablet/laptop/computer/public device/Xbox®/no preference)	120/30/12/8/14/3/34	88/14/6/10/12/16/45	X^2^(N = 412, df = 6) = 21.47, *p =* 0.002
Prevalent place of gaming (home/school/internet café/friends/no preference)	195/4/9/4/9	134/4/23/4/26	X^2^(N = 412, df = 4) = 23.63, *p <* 0.001;M: internet cafe and no preference; f: at homes

*T, trivial effect size; S, small effect size*.

### Defining the Sample Size

We followed Hair et al. (30), who recommend a sample size between 250 and 400 for validation studies and factor analyses.

### Measurements

#### Sociodemographic Information and Gaming-Related Information

Participants reported their age (in years), gender, and average gaming time per day (in hours). Further, they reported their educational level (compulsory school; high school diploma; higher education), the current job position (unemployed; employed; student; worker; free-lancer), fathers' addiction behavior (yes vs. no), the prevalent device of gaming (mobile; tablet; laptop; public device; Xbox® no preference), and the prevalent place of gaming (home; school; internet café; with friends; no preference).

### General Notes to the Translation Process

When a Persian version of a questionnaire was not available, questionnaires were translated according to the following procedure (31, 32): two translators independently translated the items into Persian. Second, the two versions of translated items were compared. In the case of complete linguistic and semantic overlap, the translation was retained. When linguistic and semantic overlaps were low, a third translator endeavored to find the best linguistic and semantic fit between divergent translations. Two independent translators then back-translated the Persian versions into English. In the case of high linguistic and semantic overlap between the original English items and the back-translated version, the Persian items were accepted as the final versions. In the case of linguistic and semantic differences, both the Persian and the translated English version were adapted until high linguistic and semantic overlap was achieved.

#### Game Addiction Scale 21 (GAS 21)

Lemmens et al. (33) developed and validated the Dutch self-rating scale to measure computer and videogame addiction. The scale consists of 21 items loading on the following seven dimensions: Salience (3 items such as: “Did you think about playing a game all day long?”), tolerance (3 items such as: “Did you play longer than intended?”), mood modification (3 items such as: “Did you play games to forget about real life?”), relapse (3 items such as: “Were you unable to reduce your game time?”), withdrawal (3 items such as: “Have you felt bad when you were unable to play?”), conflicts (3 items such as: “Have you lied about time spent on games?”), and problems (3 items such as: “Did you feel bad after playing for a long time?”). Answers are given on 5-points Likert scales ranging from 1 (= never) to 5 (= very often), with higher sum scores reflecting more problematic and addictive gaming behavior. The GAS 21 has also been validated in Portuguese (34) and Turkish (35) (Cronbach's alpha = 0.92).

#### Game Addiction Scale; 7-Items (GAS 7)

In addition, we used the short form of the scale, with seven items covering the following: preoccupation about gaming duration, tolerance, escaping from reality, unsuccessful attempts to reduce or to stop gaming, withdrawal when abstaining from gaming, deceiving others and lying on gaming duration, and continue to game despite problems. Lin et al. (18) have psychometrically validated the Persian version that we used. A French version for adolescents (36), and French and German versions for adults (37) are also available. Cronbach's alpha of the present data: 0.93.

#### Young Internet Addiction Test (YIAT)

Participants completed the Persian Young Internet Addiction Test (YIAT) (38). It consists of 20 items; typical items are: “Do you snap, yell, or act annoyed if someone bothers you while you are online?”, or “Do you choose to spend more time online rather than going out with others?”. Answers are given on 6-point Likert scales, with the following anchor points: 0 (= not at all) to 5 (= always), and with higher scores reflecting a more pronounced internet use (Cronbach's alpha = 0.90).

#### Generalized Self-Efficacy Scale (GSES)

To assess self-efficacy, the General Self-Efficacy Scale was used (39). The questionnaire consists of ten items; typical items are: “I can always manage to solve difficult problems if I try hard enough.”; “I can solve most problems if I invest the necessary effort.” Answers are given on 4-point Likert scales ranging from 1 (= not at all true) to 4 (= exactly true), with higher scores reflecting a higher self-efficacy (Cronbach's alpha = 0.88).

### Statistical Analysis

First, Items of the GAS-21 were submitted to structural equation modeling, taking into consideration the cut-off criteria proposed by Hu and Bentler (40). Next, the same GAS-21 items were submitted to exploratory factor analysis (see details in the text), as the minimum requirements to perform a factor analysis were met (30). Next, Pearson's correlations were computed between scores on the GAS-21, GAS-7, Generalized Self-efficacy, Internet Addiction Test, age (years), and mean duration of internet use (hours per day). Gender differences in scores on the GAS-21, GAS-7, the Internet Addiction Test and the General Self-efficacy were compared by *t-*tests. Mean differences in scores on the questionnaires as a function of educational level were examined by ANOVA. A *p* < 0.05 was considered as statistically significant. Structural Equation Modeling were performed with AMOS® (IBM Corporation, Armonk NY, USA); all other statistical analyses were executed using SPSS 25.0 (IBM Corporation, Armonk NY, USA) for Apple Mac®.

## Results

### General Information

Sociodemographic characteristics and gaming-related information of the 412 participants are presented in Table 1.

Overall, 412 individuals participated at the study; mean age was 21.66 years; on average, they were gaming for about 4.31 h per day; and the highest educational level was either the compulsory school or a high school diploma. The majority of participants was either employed, student, worker, or free-lancer; just 42 were unemployed. The majority was gaming on the mobile phone, while tablets, laptops, (desk) computers, public devices, or Xbox® were rarely used. Further, the majority was gaming at home, in an internet café, or had no preferences.

As regards gender comparisons, no gender differences were found for age, gaming time, BMI, highest educational level, and the current job position. Compared to male participants, female participants used less the mobile phone or had no specific preference. Further, female participants preferred gaming at home.

### Model Fit of the Initial 21 Items on the Initial Seven Factors

**Figure 1:**
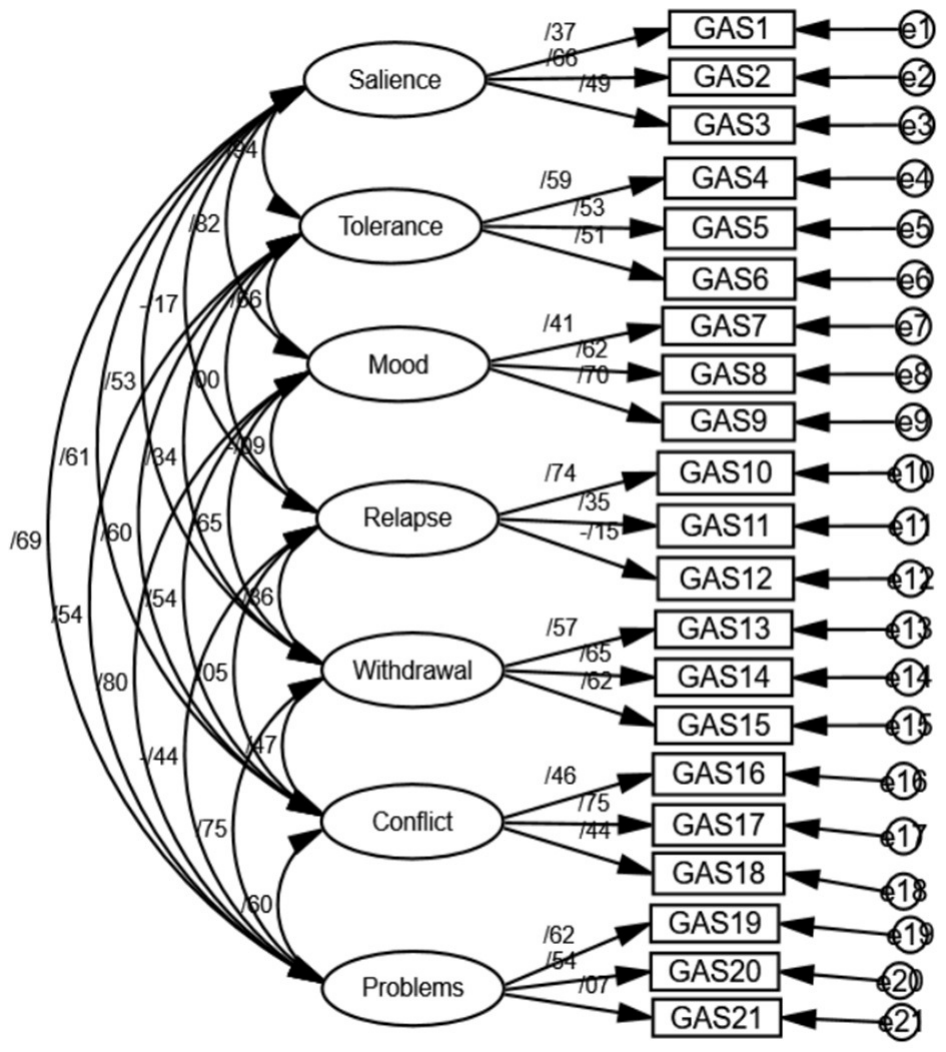
Structural equation model of the 21 items loading on the initial seven factors.

The model fit of the initial 21 items loading on the initial seven factors was satisfactory: NFI: 0.91 [expected: ≥ 0.90]; CFI: 0.95 [expected: ≥ 0.90]; AGFI: 0.91 [expected: ≥ 0.90]; GFI: 0.94 [expected: ≥ 0.90]; RMSEA: 0.053 [expected: ≤ 0.08]; X^2^(N = 412; df = 118; df = 0.11) = 8.03, *p* < 0.05 (see Figure 1).

### Factor Analysis of the GAS-21 Items

A factor analysis was performed on the items of the GAS-21. Four statistical steps were taken. First, the minimum requirements for factor analyses were met: (a) the minimum sample size of 100 participants was exceeded; (b) all items were scored in the same direction; (c) correlations were high and significant; (d) the Kaiser-Meyer-Olkin (KMO) Measure of Sampling Adequacy was acceptable at 0.838 > 0.05; (e) Bartlett's Test of Sphericity was significant [X^2^(N = 412; df = 120) = 1231.62, *p* < 0.0001]. Second, factors were extracted with Principal Component Analysis (PCA). Third, factors with Eigenvalues > 1 were extracted. Last, orthogonal varimax rotation was employed.

The first exploratory factor analysis yielded three factors with Eigenvalues > 1, together explaining 50.5% of the total variance. Next, two items loading simultaneously on two or more factors were deleted as were three items with factor loadings lower than 0.50 (item 4, Tolerance: playing longer than intended; item 7, Mood: forgetting about real life; item 10: Relapse: unable to reduce time; item 16, Conflicts: fights with others; item 20: Problems: neglected important activities). The second exploratory factor analysis this time yielded four factors with Eigenvalues >1, this time explaining 66.36% of the total variance. The factors were labeled as follows: 1. Withdrawal (36.02% of total variance); 2. Feelings of guilt and addition (14.29% of total variance) 3. Mood modification (11.15% of total variance); 4. Issues with time budget (4.90% of total variance). Factor loadings and items are presented in Table 2.

**Table 2:** Items of the Gaming Addiction Scale 21 and their factor loadings.

	**Withdrawal**	**Feelings of guilt and addiction**	**Mood modification**	**Issues with time budget**
GAS15 WITHDRAWAL feeling stressed when unable to play	0.762			
GAS13 WITHDRAWAL feeling bad when unable to play	0.656			
GAS14 WITHDRAWAL feeling bad when unable to play	0.647			
GAS12 RELAPSE failed to reduce time	0.558			
GAS5 TOLERANCE increasing amount of time		0.659		
GAS3 SALIENCE feeling addicted		0.633		
GAS6 TOLERANCE unable to stop once started		0.590		
GAS1 SALIENCE playing all day long		0.561		
GAS21 PROBLEMS feeling bad after playing a long time		0.532		
GAS2 SALIENCE much free time on gaming			0.669	
GAS9 MOOD MODIFICATION unable to stop			0.661	
GAS8 MOOD MODIFICATION gaming to release stress			0.625	
GAS19 PROBLEMS sleep deprivation				0.508
GAS18 CONFLICT lied about duration				0.775
GAS11 RELAPSE angry when unable to play				0.581
GAS17 CONFLICT neglected others				0.550

Table 3 provides the comparisons between the seven factors of the original scale and the four factor of the present computations.

**Table 3:** Comparisons between the factors of the original Game Addiction Scale 21 and the present factors.

**Original factors of the GAS 21**	**Factors of the present study**
Salience	*Wasting time and Stress release*
Tolerance	Feeling addicted
Mood modification	*Wasting time and Stress release*
Relapse	Relapse
Withdrawal	Withdrawal
Conflicts	*Neglect of duties and other persons* *Social conflicts*
Problems	General problems

*GAS, Game Addiction Scale 21. Factors in italics: factors of the present study with low or double overlap with the original factors*.

### Correlations Between Scores of the GAS-21, GAS-7, the Young Internet Addiction Test (YIAT), the Generalized Self-Efficacy Scale (GSES), Age (Years), and Gaming Duration (Hours/Day)

Table 4 reports the correlation coefficients between the scores on the questionnaires, age, and gaming duration per day.

- Higher GAS-21 scores were related to higher GAS-7 scores, higher GSES scores (though, the correlation was weak), higher YIAT scores, lower age and longer gaming duration per day.
- Higher GAS-7 scores correlated with lower YIAT scores, lower age, and longer gaming duration per day. GAS-7 scores were unrelated to GSES scores.
- Higher GSES scores were related to higher YIAT scores and lower age; GSES scores were unrelated to gaming duration per day.
- Higher YIAT scores were related to a longer gaming duration per day, but unrelated to age.
- Higher age was associated with shorter gaming duration per day.

**Table 4:** Correlations between scores of the Gaming Addiction Scale (GAS-21), the Gaming Addiction Scale—Short Form (GAS-7), the Internet Addiction Test (IAT), the General Self-efficacy (GSE), and age, and gaming duration per day.

			**Dimensions**				
		**1**	**2**	**3**	**4**	**5**	**6**
1	Game Addiction Scale 21	–	0.57^***^	0.10^*^	0.39^***^	−0.21^***^	0.29^***^
2	Game Addiction Scale 7		–	0.00	0.26^***^	−0.21^***^	0.30^***^
3	Generalized Self-efficacy Scale			–	0.13^***^	0.21^***^	0.07
4	Young Internet Addiction Test				–	−0.04	0.26^***^
5	Age (years)					–	−0.17^***^
6	Gaming duration (h/day)						–

* *p < 0.05*;

*** *p < 0.001*.

## Discussion

The key findings of the present study were that this Persian version of the Game Addiction Scale 21 is a suitable and psychometrically sound questionnaire for assessing an individual's tendency to display excessive internet gaming behavior. Given the wide age range of the participants—from adolescence to adulthood—the questionnaire appears to be appropriate irrespective of age. Thus, the questionnaire can be understood as a transgenerational tool. Internal psychometric properties were satisfactory, while external validity was modest. Further, the solution with 16 items loading on four factors appears respond to the ecological need of parsimony.

The DSM-5 has tentatively identified pathological internet gaming as a disorder (10); here, excessive and pathological internet gaming (both online or offline) is understood as behavior characterized as addictive. Specifically, this involves display of following behaviors over the previous 12 months such as preoccupation with gaming, the need to spend more time gaming to satisfy the urge, loss of interest in previously enjoyed activities due to gaming, the use of gaming to relieve negative moods, along with the risk, having jeopardized or lost a job or relationship due to gaming (see also Introduction).

The GAS 21 is a self-rating tool to assess pathological internet gaming. From the 21 items, the original tool extracted the following seven factors: salience, tolerance, mood modification, relapse, withdrawal, conflicts, and problems (33–35). Similarly, in the present analysis, four factors were extracted (see Tables 2, 3). These factors were: Withdrawal, Feelings of guilt and addiction; mood modification, and Issues of time budget.

Next, the GAS 21 overall score was moderately correlated with the Young Internet Addiction scale, negatively with age, and positively with daily gaming duration (see Table 3).

Overall, this pattern of results was expected; internet gaming addiction and internet addiction are expected to share a part of their variance. It seems likely that internet gaming addiction is associated with longer gaming duration per day. Similarly, lower age was associated with higher internet gaming addiction. This latter result accords with the pattern reported by Mihara and Higuchi (19) in their meta-analysis; they found that across adolescence and young adulthood, younger age was associated with higher internet gaming addiction scores.

For the following reasons, we would therefore emphasize that children and adolescents need particular protection against this addiction. First, Sugaya et al. (41) noted that minors are susceptible to problematic internet gaming use, most probably due to age-related underdevelopment of cognitive control. Second, findings from MRI-studies (20–22) have shown that the prefrontal cortex undergoes substantial morphological changes across adolescence and into early adulthood. At a behavioral level, such substantial morphological changes might be associated with lower cognitive control, lower impulse inhibition, and less emotion regulation. Third, we note that 55.3% of participants were females. Following Hyde et al. (42) research on major depressive disorders among women showed that the following factors may contribute to higher depression rates among women, compared to men: These factors are affective (emotional reactivity), biological (genetic vulnerability, pubertal hormones, pubertal timing, and development), and cognitive (cognitive style, objectified consciousness, rumination) factors, which in their additive fashion may contribute to a higher vulnerability to depression. Further, vulnerability is increased when negative life events interfere. Overall, it is conceivable that these factors may have biased above all female participants' pattern of results.

**As regards self-efficacy and contrary to our expectations, the GAS overall score was positively associated with self-efficacy, though, as explained in more details below, the correlation coefficient of r** **=** **0.10 was trivial. The data available from the study is unable directly to clarify why this positive association emerged. We therefore advance what is necessarily a rather speculative interpretation: 1. Regular gaming implies that the individual becomes skilled and successful; otherwise, one would quit gaming; thus, it is possible that players associate their success with their own capacities, and thus with their own (self-) efficacy. If this is the case regular to excessive gaming could be associated with higher self-efficacy. 2. The correlation coefficient was r** **=** **0.10, and inspection of the scatterplot did not reveal subgroups or non-linear associations which could have obscured the linear association. Nonetheless, the scatter plot does indicate that for some participants gaming addiction scores and self-efficacy scores were negatively associated, while for other participants gaming addiction scores and self-efficacy scores were positively associated. This suggests that some participants might have related the self-efficacy items to the more general circumstances of their current lives (probably producing a negative association), while other participants might have related the self-efficacy items to their gaming skills (probably producing a positive association). The overall effect of pooling the positive and negative associations between gaming addiction and self-efficacy would then have been to blur the general pattern. While no conclusive answers can be derived from the present study, an implication is that future studies might employ more specific self-efficacy questionnaires**.

The novelty of the results should be balanced against the following limitations. First, an assessment of symptoms of depression, anxiety and obsessive behavior would have helped to extend our understanding of the possible psychopathological mechanisms of gaming addiction. Second, a related possibility is that expert ratings based, for example, on a thorough clinical interview for DSM-5 psychiatric disorders (43) could have shed further light on the underlying psychopathology. Third, we assessed exclusively individuals self-identified as having “issues with gaming”; thus, the sample itself might be biased, and assessing individuals self-identifying as having no issues with gaming might have yielded different factor solutions and perhaps a more nuanced pattern of results. Fourth, for adolescents, it would have been interesting to associate gaming addiction scores with educational outcomes. This would have allowed the evaluation of the extent to which different dimensions of gaming addiction adversely affected performance at school. Fifth, the corresponding interesting question for adults is to what extent dimensions of gaming addiction were associated with performance in the work place. Sixth, it is possible that other latent, but unassessed psychological dimensions might have biased two or more dimensions in the same or opposite directions. Seventh, it would have been interesting to know if participants with high scores on internet gaming also scored highly for other addictive behaviors such as substance use (e.g., alcohol, tobacco, cannabis) or other pathological behaviors such as excessive sports activity, or gambling. Eighth, assessments of emotion regulation and cognitive control could have shed light on possible dysfunctional cognitive and emotional processes, this is to say: It is highly conceivable that excessive gaming had the function to cope with stress, and to reduce unpleasant feelings and lower impulse control. In this view, we note that impulsivity and lower cognitive control are core features of addiction susceptibility among adolescents with sleep disorders (44) and of attention-deficit/hyperactivity disorder (ADHD). Given that the prevalence rate of ADHD is about 5.6% in children and adolescents, and about 2.6% in adults [see Zamani Sani et al. (45) for a brief overview], statistically, 17–23 participants should report clear signs of ADHD. Ninth, a thorough assessment of pubertal stages (for adolescent participants) and skills on emotion regulation, along with biomarkers such as cortisol or testosterone secretion both cross-sectionally and longitudinally would have allowed to run an in-depth investigation to identify both underlying psychological and biological predictors and consequences of internet gaming addiction behavior. Tenth, we were able to test the construct validity with the Farsi version of the GAS-7 for adolescents (18); the correlation coefficient was r = 0.57; this means that the variance of the GAS-7 scores could explain 32.49% of the variance of the GAS-21 scores. We note that the GAS-7 was psychometrically tested for adolescents, while in the present study we crossed the borders of developmental stages (26) to focus on problematic behavioral addiction disorder as an age-independent behavior. Last, Király et al. (46) presented the psychometric validation of the Ten-Item Internet Gaming Disorder Test (IGDT-10), including also a large sample of Iranian participants (*n* = 791). When running the present study, the IGDT-10 was not available to us; otherwise, we would have used this measurement for further cross-evaluations of the present data.

## Conclusion

The Persian version of the GAS 21 is a suitable and easily completed questionnaire assessment of internet gaming addiction behavior. Further, the solution with 16 items loading on four factors appears respond to the ecological need of keeping a measurement as short and parsimonious as possible. Last, along with internet addiction, gaming addiction is potentially becoming a serious psychological health problem, for both the individual and wider society.

## Data Availability Statement

The raw data supporting the conclusions of this article will be made available by the authors, without undue reservation.

## Ethics Statement

The studies involving human participants were reviewed and approved by the Medical Research and Ethical Committee of Kermanshah University of Medical Sciences, Kermanshah, Iran with registration No. Kums.REC.1396.479 (November 11, 2017). Written informed consent to participate in this study was provided by the participants' legal guardian/next of kin.

## Author Contributions

NA: conceptualization, formal analysis, data curation, project administration, wrote: draft, final version, supervision. VF: conceptualization, project administration, wrote: draft, final version, supervision. MA: conceptualization, formal analysis, data curation, project administration, wrote: draft, final version, supervision. DS-B: conceptualization, formal analysis, wrote: draft, final version, supervision. KMD: conceptualization, formal analysis, data curation, project administration, wrote: draft, final version, supervision. ME: conceptualization, formal analysis, data curation, project administration, wrote: draft, final version, supervision. AB: conceptualization, formal analysis, data curation, project administration, wrote: draft, final version, supervision. SB: conceptualization, formal analysis, data curation, project administration, wrote: draft, final version, supervision. All authors contributed to the article and approved the submitted version.

## Conflict of Interest

The authors declare that the research was conducted in the absence of any commercial or financial relationships that could be construed as a potential conflict of interest.
